# Modulating organelle distribution using light-inducible heterodimerization in *C. elegans*

**DOI:** 10.1016/j.xpro.2020.100273

**Published:** 2021-01-13

**Authors:** Tobias B. Dansen, Sasha De Henau

**Affiliations:** 1 Center for Molecular Medicine, Molecular Cancer Research, University Medical Center Utrecht, Universiteitsweg 100, 3584 CG Utrecht, the Netherlands

**Keywords:** Cell biology, Microscopy, Model organisms

## Abstract

The relative positioning of organelles underlies fundamental cellular processes, including signaling, polarization, and cellular growth. Here, we describe the usage of a light-dependent heterodimerization system, LOVpep-ePDZ, to alter organelle positioning locally and reversibly in order to study the functional consequences of organelle positioning. The protocol gives details on how to accomplish expression of fusion proteins encoding this system, describes the imaging parameters to achieve subcellular activation in *C. elegans*, and may be adapted for use in other model systems.

For complete details on the use and execution of this protocol, please refer to De Henau et al. (2020).

## Highlights

• Design guidelines for constructs for LOVpep-ePDZ-based organelle translocation
• Generation of constructs for stable integration in *C. elegans*
• Optimization of conditions for light-induced activation of the LOVpep-ePDZ system
• Troubleshooting of the most common pitfalls associated with the above procedures

## Graphical Abstract

**Figure:**
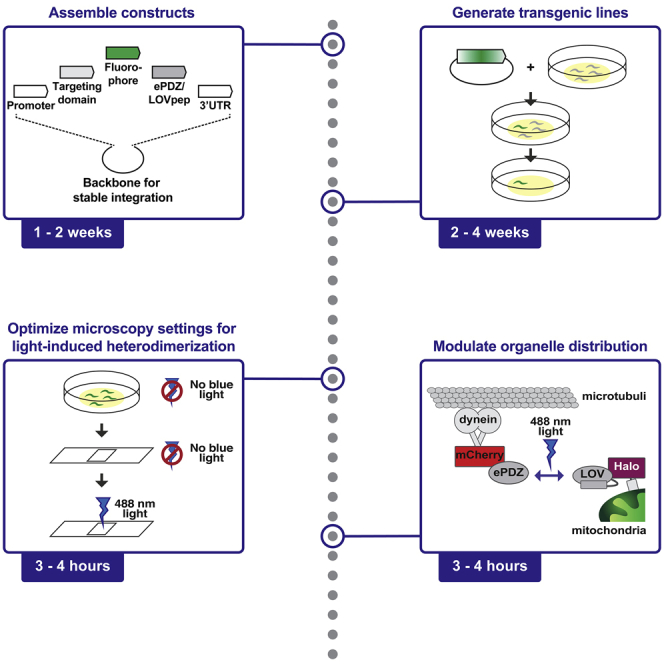

## Before you begin

Manipulation of subcellular organelle positioning can be achieved using the light-dependent heterodimerization system LOVpep-ePDZ, an example of an optogenetic approach, in which a photosensitive LOVpep domain binds an engineered PDZ domain (ePDZ) after exposure to blue light (<500 nm) (Strickland et al., 2012). In the following, we describe how to implement and use this system. While we focus on the application of this system in the *Caenorhabditis elegans* zygote, most principles described here are likely adaptable to any transparent biological model system or organism.

**Figure 1:**
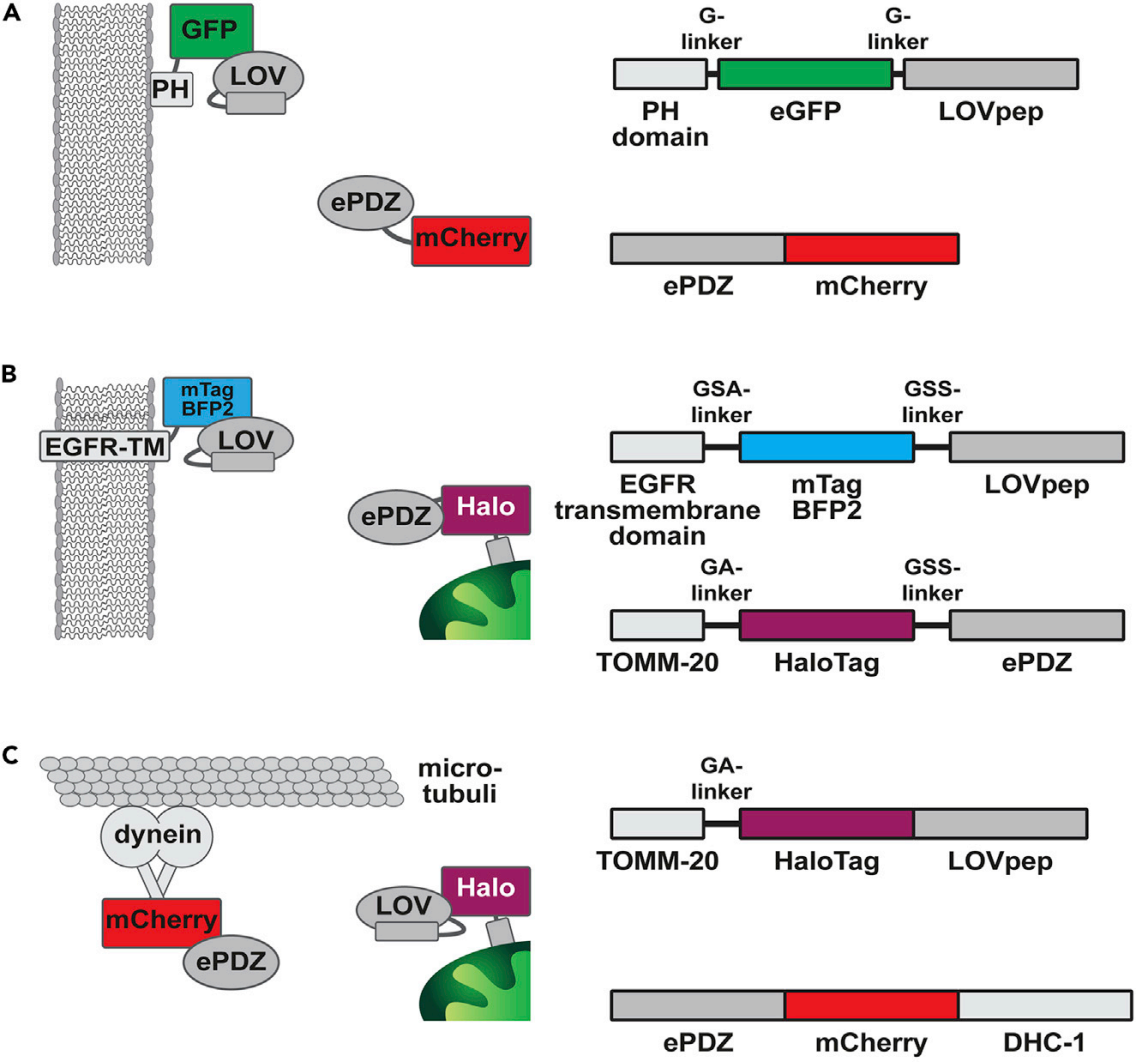
Design of light-inducible LOVpep-ePDZ heterodimerization modules (A) Scheme for the induced recruitment of ePDZ::mCherry to membrane bound PH::eGFP::LOV and underlying construct design. (B) Scheme for the induced trapping of mitochondria with TOMM20::HALO::ePDZ to membrane EGFR-TM::mTagBFP2::LOV and underlying construct design. (C) Scheme for the induced transport of mitochondria with TOMM20::HALO::LOVpep via dynein heavy chain fused to ePDZ and mCherry, together with underlying construct design.

To set up this system, plasmids encoding LOVpep and ePDZ need to be generated, where one is fused to an organelle targeting sequence and the other to a protein domain that localizes to the desired site for organelle relocation (Figure 1). Subsequent stable integration of these constructs is preferred: this allows the relative concentrations between dimerizing proteins to be more consistent, which improves the efficiency of light-induced activation (Krishnamurthy et al., 2016). Stable integration is also essential for expression in the *C. elegans* germline and early embryo. However, transient expression can be useful for rapid screening of functional constructs. In the following, we describe how to design and generate the constructs encoding the LOVpep-ePDZ system.

***Note:*** Besides LOVpep-ePDZ, a number of other light-inducible protein dimerization techniques have been developed. The binding affinity and activation and reversion kinetics of these light-inducible protein dimerization systems are important parameters to consider: systems with a tighter affinity are more suited to induce a fast functional response. However, it also makes them more sensitive to residual dark-state binding. For multiday experiments, minimal dark-state binding seems crucial to avoid unwanted background activation and perturbation of the system. The activation and reversion kinetics in turn determine how frequently the system needs to be exposed to maintain dimerization, and faster kinetics allow for more temporal resolution (Hallett et al., 2016; Pathak et al., 2014; Strickland et al., 2012).

The LOVpep-ePDZ (also referred to as TULIP) used here has a reported in vitro affinity of 72 μM in the dark and 12 μM under blue light, for a 6-fold change (Hallett et al., 2016). However, while ePDZ can be labeled N- and C-terminal, LOVpep does not tolerate C-terminal fusions.

iLID-SspB is mechanistically related to LOVpep-ePDZ, is also activated by blue light, and comes in three variations: nano (4.7 μM dark/ 0.13 μM blue light), micro (47 μM dark/ 0.8 μM blue light) and milli (125 μM dark/ 3 μM blue light), for at least a 25-fold change in all three pairs (Guntas et al., 2015; Zimmerman et al., 2016). However, the tighter affinity of the iLID-SspB pairs compared to LOVpep-ePDZ appear to make it more susceptible to dark-state binding. Both iLID and SspB tolerate N- and C-terminal fusions. LOVpep-ePDZ and iLID-SspB show comparable activation and reversion kinetics.

A third example of a blue-light-inducible dimer is the CRY2-CIB1 pair. Rather than an intrinsic change in affinity upon blue light stimulation, it appears it is the light-induced homo-oligomerization of CRY2 that drives colocalization with CIB1 (Bugaj et al., 2013; Hallett et al., 2016; Kennedy et al., 2010; Lee et al., 2014). Because of this, orientation-specific tagging effects of either component need to be considered when implementing this system. Compared with the previous two methods, CRY2-CIB1 shows slower activation and reversion kinetics (Hallett et al., 2016).

A final example of a light-sensitive heterodimerization system is the Phy/Pif pair, which uses a different light spectrum than the three previous methods: this pair forms under red light and dissociates in darkness or under far-red illumination. Compared to the previous systems, it shows higher fold levels of activation and low background binding. This method is limited in that it requires the external addition of the cofactor phycocyanobilin, which is not always straightforward (Adrian et al., 2017; Levskaya et al., 2009; Pathak et al., 2014).

A direct comparison of these systems, which allows for a better understanding of their unique advantages and limitations, can for example be found in Hallett et al. (2016) and Pathak et al. (2014). We advise the reader to carefully assess the requirements of their own light-dependent heterodimerization experiments before deciding on which system to use.

### Design the plasmids containing the LOVpep and ePDZ constructs

**Timing: 1–4 h**

1. Choose sequences encoding proteins or protein domains that connect LOVpep and ePDZ to the organelle of interest and to the protein that will be used to relocalize the organelle to the desired site respectively (Figure 1). To achieve local activation, it is critical that diffusion of activated LOVpep outside of the activated region is limited as much as possible. To this end, LOVpep is tagged to the least dynamic structure of the two. For example, to transport mitochondria along microtubules we fused LOVpep to the relatively immobile mitochondria using TOMM-20 and fused dynein heavy chain DHC-1 to ePDZ (Figure 1C) (Fan et al., 2019). On the other hand, to trap mitochondria at the cell membrane we fused LOVpep to a transmembrane domain to localize it to the plasma membrane, while ePDZ was now fused to the relatively more mobile mitochondria using TOMM-20 (Figure 1B) (De Henau et al., 2020).
2. ePDZ can be placed either N- or C-terminal of the protein (domain) of choice in the final construct. On the other hand, LOVpep can only be placed at the C terminus of the final construct. LOVpep interacts with ePDZ via a C-terminal Jα-helix, which needs to remain free to maintain its functionality (Hallett et al., 2016; Strickland et al., 2012).
3. Ideally, label both constructs with fluorophores to ensure proper expression and localization. Keep in mind here that LOVpep is traditionally activated using 470–500 nm light (Harper et al., 2003; Strickland et al., 2012) but is also significantly activated by shorter and slightly longer wavelengths. For example, we observed light-induced LOVpep-ePDZ heterodimerization using an excitation power of 4.8 μW 405 nm laser light, 0.002 μW 458 nm laser light and 0.005 μW 514 nm laser light (all 5 iterations and pixel dwell time of 8 μs, exposure every 2 s). These settings are considerably lower than what we normally use to excite blue, green, and yellow fluorophores. Therefore, in case the LOVpep or ePDZ constructs need to be visualized and followed during light-activation experiments, one cannot use fluorophores that require excitation with wavelengths between 400 and 520 nm, such as mTAGBFP2, eGFP and Venus (Hallett et al., 2016). Excitation of fluorophores with an excitation spectrum that is distinct from the excitation wavelength of the LOVpep domain, such as mCherry, mScarlet and HaloTag combined with the JF_646_ HaloTag ligand (Grimm et al., 2015), do not induce LOVpep-ePDZ dimerization. When it is not required to visualize LOVpep or ePDZ during light-activation experiments, you can tag them with mTAGBFP2 or eGFP to ensure proper expression. This way, higher wavelengths remain free for visualization of other proteins or biological processes.
4. It is recommended to use suitable linkers (such as GGSGGSGGS or GAGAGAGAGAGA) between the LOVpep, ePDZ, and the protein domains of choice to allow for proper protein folding.
5. LOVpep-ePDZ is sensitive to differences in expression levels because it is restricted to a 6-fold increase in dimerization affinity upon illumination. It is therefore recommended to use identical or comparably strong regulatory regions (promoter, 3′UTR) to drive expression. When going for single-copy integration techniques, aim for regulatory regions that cause moderate-strong protein expression, in order to achieve LOVpep and ePDZ levels that are sufficiently high to permit light-depending LOVpep-ePDZ interactions. We successfully used *mex-5* promoter or *fbf-1* promoter in combination with *tbb-2* 3′UTR (sequences and expression patterns detailed in Merritt et al. (2008)) to achieve LOVpep and ePDZ germline expression that is compatible with light-depending activation (De Henau et al., 2020; Fan et al., 2019).
6. Once the transgene is designed, codon optimization for expression in *C. elegans* (Redemann et al., 2011), or the model organism of choice, is advised. To specifically increase expression in the *C. elegans* germline and avoid germline silencing, germline-specific codon optimization is available and highly recommended (Fielmich et al., 2018). Additional approaches to avoid germline silencing are the removal of homology to piRNAs (Batista et al., 2008) and introduction of PATC introns (Frokjaer-Jensen et al., 2016).

**CRITICAL:** Make sure that the final ePDZ and LOV constructs show an overlapping subcellular expression pattern, so that upon light activation LOVpep is capable of binding ePDZ. When using motor proteins, make sure that they are active in the cell of interest. Alternatively, use constitutively active or inducible active motor proteins (Nijenhuis et al., 2020). Also make sure the cell of interest has the cytoskeletal structures that are required for the motor proteins to relocate the organelle of interest.

### Assemble and integrate the plasmids carrying LOVpep and ePDZ constructs

**Timing: 2 weeks**

Assembling the LOVpep and ePDZ constructs requires combining multiple genetic elements. Modular cloning techniques such as Gateway (Walhout et al., 2000), Gibson (Gibson et al., 2009) and Golden Gate (Engler et al., 2009) are preferred, given that they are faster and hence more favorable for screening for functional constructs. New constructs for photo-inducible heterodimerization do not always result in the expected organelle manipulation and might for example suffer from high levels of dark-state heterodimerization or low efficiency light-induced heterodimerization. It might therefore be necessary to test alternative organelle adaptors, relocalization proteins, mutants of LOV-ePDZ with different affinity properties (Strickland et al., 2012) or even different light-inducible heterodimerization systems (Adrian et al., 2017; Hallett et al., 2016; Nijenhuis et al., 2020) before the desired experimental setup is achieved.

To stably introduce transgenes in *C. elegans*, we recommend generating plasmids that are compatible with the Mos1-mediated Single-Copy Insertion (MosSCI) method (Frokjaer-Jensen et al., 2012; Frokjaer-Jensen et al., 2008; Frokjaer-Jensen et al., 2014). This method creates single-copy transgenes at defined positions within the genome, which facilitates subsequent crossing of the transgenes into a single line, generates comparable expression levels of the transgenes and decreases the probability of germline silencing. Alternatively, CRISPR/Cas9 mediated gene editing (Nance and Frokjaer-Jensen, 2019) can be used when endogenous proteins need to be tagged.

**Figure 2:**
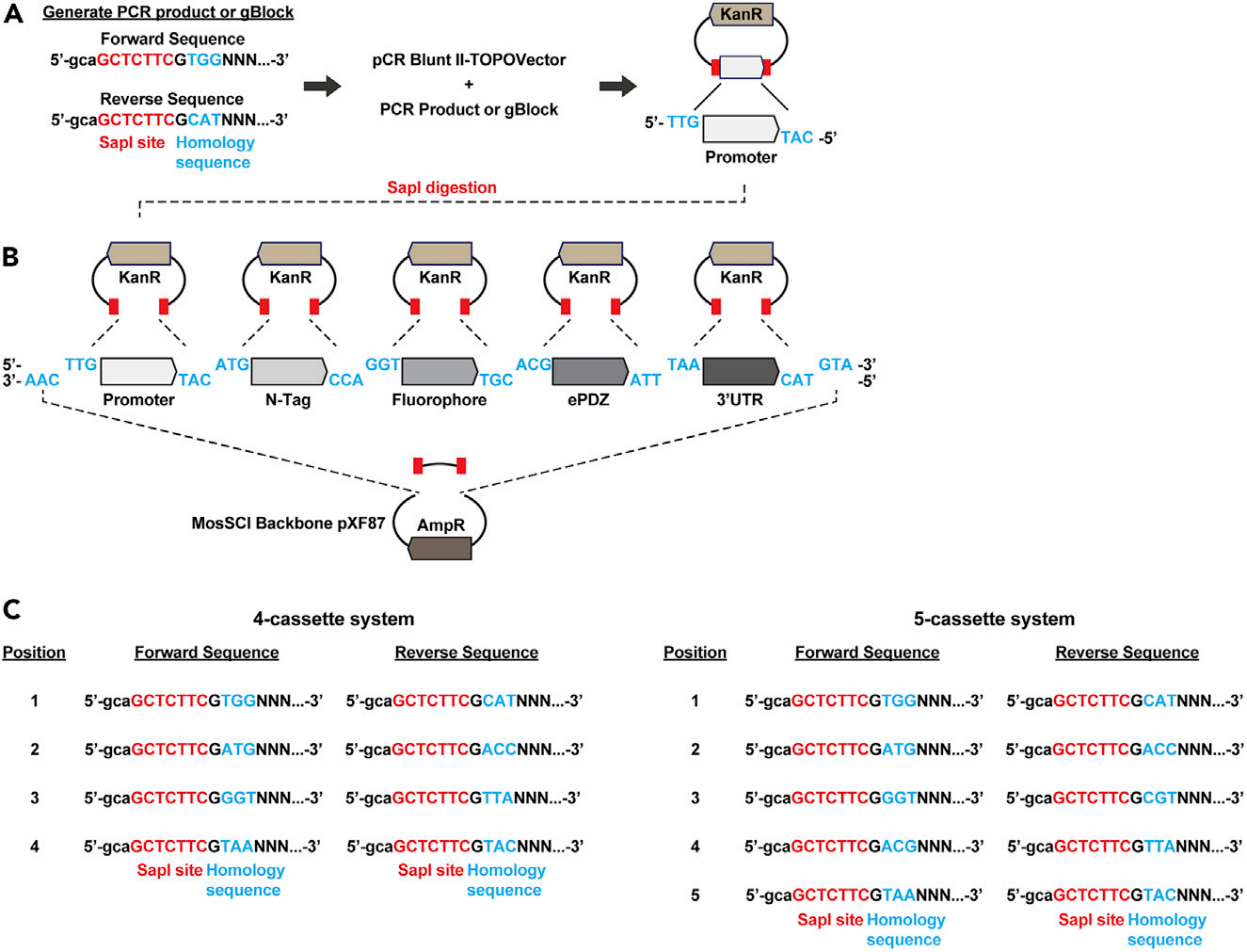
The modular Golden Gate cloning technique SapTrap to assemble *C. elegans* MosSCI transgene vectors (A) Gene fragments are cloned into the pCR-Blunt II-TOPO Vector to create donor cassette plasmids. Note that gene fragments are flanked by a SapI recognition site (red) and homology sequences (blue) that define the position in the final MosSCI transgene vector. (B) Modular assembly of the MosSCI transgene vector. Individual gene fragments are liberated from the donor cassettes by SapI digestion, while the 5′-homology sequences define the position of the gene fragments in the final MosSCI transgene vector. Note that the SapI recognition sites are oriented such that upon digestion they are removed from the individual gene fragments and vector backbone. (C) Overview of primer design for SapTrap, for the 4 cassette or 5 cassette system.

With the above in mind, we recommend the recently developed Golden Gate cloning technique SapTrap to assemble *C. elegans* MosSCI transgene vectors (Fan et al., 2019) carrying LOVpep and ePDZ constructs in a fast, efficient, inexpensive, and scar-free manner (Figure 2). The MosSCI backbone and donor plasmids carrying LOVpep and ePDZ that are compatible with this method are available at Addgene (Fan et al., 2019). In the following we detail how MosSCI transgene vectors using the SapTrap method can be generated.

7. Find templates encoding the parts you want to combine and order primers to produce gene fragments flanked by SapI restriction sites. Key in the SapTrap method is that SapI cuts DNA at defined positions adjacent to its recognition sequence to generate three-base 5′ overhangs (Figure 2, red sequence/bars=recognition site, blue sequence/bars= three-base 5′ overhangs). By designing SapI restriction fragments with complementary overhangs, multiple fragments can be assembled together in a defined order in a single digestion and ligation reaction (Figure 2B, the defined order is illustrated by the fragments ranging from light gray to dark gray). Primer design and examples of primers can be found in Figure 2C and in the study by Fan et al. (2019). If templates are not available, codon-optimized sequences can be ordered. We order sequences as gBlocks (Integrated DNA Technologies).
8. When you amplify your gene fragments with your primers and template DNA, use a high-fidelity PCR kit as per the manufacturer’s instructions.
9. Generate donor plasmids by cloning the PCR products or gBlocks into the pCR BluntII vector backbone using the Zero Blunt Topo system (Thermo Fisher Scientific), as per the manufacturer’s instructions (Figure 2A).
10. Transform the Zero Blunt Topo assembly reaction into chemically competent cells: Thaw 50 μL competent cells on ice. Still on ice, add 5 μL of assembly reaction to the cells and incubate for 20–30 min. Heat shock the cells at 42°C for 30 s. After transformation and before plating, add 0.2 mL of 18°C–24°C SOC Medium and shake at 225 rpm 37°C for 1 h. Spread the cells onto pre-warmed kanamycin-selective plates and incubate 16–20 h at 37°C.

**Pause point:** Bacterial colonies can be stored at 4°C for up to 2–3 weeks.

11. Pick colonies, grow 16–20 h at 37°C and extract the plasmid DNA using a miniprep kit, as per the manufacturer’s instructions. Carry out diagnostic restriction enzyme digests followed by sequencing to confirm that the donor plasmid has the correct, mutation-free insert.
12. Adjust the volume of donor plasmids to bring to a final concentration of 50 nM.
13. MosSCI targeting vectors are assembled using the SapTrap method in a single tube (Figure 2B) using the following protocol: add 1 μL 50 nM pXF87 (MosSCI backbone, available at Addgene), 1 μL 50 nM of each donor cassette plasmid, 5 μL 10× NEB cutsmart buffer, 5 μL 10 mM ATP (not dATP), 1 μL SapI enzyme (10 units), 1 μL T4 DNA ligase (400 units) and ddH_2_O to a final volume of 50 μL. Incubate this reaction mixture 5 min at 37°C (=SapI digestion) and 5 min at 16°C (ligation), repeating this for a total of 35 cycles. This is followed by a final SapI digestion step of at least 1 h and up to 16 h at 37°C to cut any remaining, unligated pXF87 backbone. After this final step, put immediately on ice and use 5 μL for transformation into chemically competent cells as described above. Spread the cells onto pre-warmed ampicillin-selective plates and incubate 16–20 h at 37°C (Troubleshooting 1, Troubleshooting 2).
14. Isolate plasmid DNA and screen for correct assembly using diagnostic restriction and sequencing. In our hands, between 10%–90% of colonies have the correctly assembled plasmids.
15. Once the MosSCI transgene vectors are made, these can be integrated at defined landing sites in the chromosome of choice by injecting the vectors into universal MosSCI strains (Frokjaer-Jensen et al., 2014). We co-inject the MosSCI transgene vector (50 ng/μL) together with a helper plasmid encoding the Mos1 transposase (50 ng/μL pCFJ601), and with three negative selection markers to select against extrachromosomal array-bearing transgenic animals (10 ng/μL pMA122, 2.5 ng/μL pCFJ90 and 5 ng/μL pCFJ104) (Frokjaer-Jensen et al., 2014). For a detailed protocol describing the microinjection procedure, please refer to Berkowitz et al. (2008).
16. Injection of 20–30 animals is in general sufficient to obtain at least one stable transgenic line. Place 3–4 injected animals per NGM plates with OP50 *E. coli* and keep them at 25°C until they are starved (7–10 days).
17. Heat-shock animals for 2 h at 34°C to activate the peel-1 toxin (encoded by pMA122) and selectively kill animals that carry extrachromosomal arrays. Using an air incubator with a fan and spreading out plates evenly ensures that plates warm up relatively fast to 34°C to efficiently induce the heat shock.
18. 4–24 h following the heat shock, screen for plates that contain animals that are alive, move well, and lack the fluorescent co-injection markers. Chunk positive plates to new seeded NGM plates. Two days later, transfer a single adult animal to a new seeded NGM plates. If possible, screen for expression of the inserted transgene (germline expression, for example) before picking a worm to set up a clonal culture.
19. In the isolated lines, verify that the transgene is expressed and localizes to the expected location. For germline expression, note that it can take 1–3 generations before germline silencing of the transgene disappears and the transgene becomes expressed (Troubleshooting 3).
20. Verify that the transgene has been correctly inserted at the chromosome of choice using the oligos described on www.wormbuilder.org (under “universal insert verification”) (Frokjaer-Jensen et al., 2014).
21. Once stable lines carrying the individual transgenes have been made, they can be crossed to generate a single strain expressing both the LOVpep and ePDZ transgenes. A detailed protocol for setting up crosses can be found in Fay (2006).

## Key resources table

**Table:** 

REAGENT or RESOURCE	SOURCE	IDENTIFIER
**Bacterial and virus strains**		

*E.coli* HST08 - Stellar Competent Cells	Clontech	Cat#636763

**Chemicals, peptides, and recombinant proteins**		

Janelia Fluor 646	Tocris	Cat#6590
Zero Blunt TOPO PCR Cloning Kit	Thermo Fisher Scientific	Cat#450245
SapI & Cutsmart buffer	NEB	Cat#R0569
T4 DNA ligase	NEB	Cat#M0202
ATP	Sigma-Aldrich	Cat#A2383
SOC medium	Clontech	Cat#636763
Kanamycin sulfate	Thermo Fisher Scientific	Cat#11815032
Ampicillin	Sigma-Aldrich	Cat#A9518

**Experimental models: organisms/strains**		

ttTi5605(he314[Ppie-1::glo-epdz::mcherry(smu-1)::tbb-2(3′UTR)]) II; cxTi10816(he259[Peft-3::ph::co-egfp::co-lov::tbb-2(3′UTR)]) IV	(Fielmich et al., 2018)	SV2061
dhc-1(he255[epdz::mcherry::dhc-1]) I; utdSi51(mex-5p::tomm-20(aa 1–55)::halotag::lov::tbb-2 3′UTR + unc119(+)) II	(Fan et al., 2019)	TBD307
he314[Ppie-1::glo-epdz::mcherry(smu-1)::tbb-2(3′UTR)] II; unc-119(ed3) III; utdSi45[mex-5p::glo-egfr-tm::glo-mtagbfp2::co-lov::tbb-2(3′UTR)] V	(De Henau et al., 2020)	TBD321
cp13[nmy-2::gfp + LoxP] I; utdSi44 [mex-5p::tomm-20::glo-epdz::glo-halo::tbb-2(3′UTR)] II; it315[mCherry::par-2]) III; utdSi45[mex-5p::glo-egfr-tm::glo-mtagbfp2::co-lov::tbb-2(3′UTR)] V	(De Henau et al., 2020)	TBD322

**Recombinant DNA**		

pXF–7 - MosSCI backbone	Addgene	Cat#139027
pXF2–6 - LOVpep domain	Addgene	Cat#139048
pSDH52 – ePDZ domain	Addgene	Cat#139049
pSDH52 – ePDZ domain	Addgene	Cat#139033
pXF87- mex-5p::tomm-20::glo-epdz::glo-halo::tbb-2(3′UTR)	(De Henau et al., 2020)	N/A
pXF87- mex-5p::PH::glo-mtagbfp2::co-lov::tbb-2(3′UTR)	(De Henau et al., 2020)	N/A
pXF87- mex-5p::glo-egfr-tm::glo-mtagbfp2::co-lov::tbb-2(3′UTR)	(De Henau et al., 2020)	N/A
pXF87- mex-5p::tomm-20(aa 1–55)::halotag::lov::tbb-2 3′UTR + unc119(+)) II	(Fan et al., 2019)	N/A

**Software and algorithms**		

ZEN version 14.0.7.201	Carl Zeiss Microscopy	https://www.zeiss.com/microscopy/int/products/microscope-software/zen.html
FiJi ImageJ Version 2.0.0-rc-63/1.51s	(Schneider et al., 2012)	https://imagej.net/Fiji
PRISM version	GraphPad	https://www.graphpad.com/scientific-software/prism/

## Materials and equipment

### Equipment

#### Confocal microscope

Images were captured using an Axioskop2 LSM880 scanning confocal microscope (Carl Zeiss) with a 40× oil objective (Plan Apochromat; numerical aperture [NA] 1.4) using Zen image acquisition software 2.3 SP1 FP3 (black) 64 bit. Power of the Argon laser (458, 488, 514 nm) was set to 488 nm 0.001%–0.01% when used for light-activation experiments, which corresponds to 0.01–0.1 μW, and to 0.5%–2% when used for eGFP imaging, which corresponds to 10–40 μW. Power of the DPSS 561-10 and HeNe633 was set between 0.5% and 3%, corresponding to 23–130 μW and 8–45 μW respectively.

### Alternates

Other microscopes are compatible as long as they can apply 488 nm laser light, and in a defined region of interest if localized light-induced activation is needed.

## Step-by-step method details

### Prepare imaging setup

**Timing: 10 min**

**CRITICAL:** Very low levels of blue light are sufficient to activate LOVpep, so all environmental blue light that could reach the animals during culturing and sample preparation needs to be eliminated as much as practically feasible.

1. Store the animals in a box that blocks environmental light, e.g., by wrapping the box in aluminum foil.
2. If the room used for sample preparation and imaging needs to be illuminated, use a light source filtered for blue light. If necessary, aluminum foil can be used to cover the microscope setup and blue light of monitors can be omitted by adjusting the hardware display settings.
3. Insert optical (orange) filters in the light paths of the dissection scope and of the imaging microscope to remove LOVpep-activating wavelengths from the transmitted light used to handle samples.

### Prepare microscope settings for global and local light-induced heterodimerization

**Timing: 3–4 h**

**CRITICAL:** The conditions used for light-induced activation need to be thoroughly optimized to ensure minimal activation before the experiment or outside the region of interest, especially since LOVpep activation requires very low light exposure.

4. To setup the microscope, use a strain in which heterodimerization is straightforward to score and where ePDZ is tagged with a red or far-red fluorophore so its distribution can be visualized without activating LOVpep.
  a. For example, a strain expressing cytosolic ePDZ combined with a membrane anchored LOVpep allows for easy scoring of membrane recruitment of ePDZ-mCherry (De Henau et al., 2020; Fielmich et al., 2018) (Figure 3A, Methods videos S1 and S2). A cytosolic ePDZ combined with an organelle anchored LOVpep works equally well (Hallett et al., 2016).

**Figure 3:**
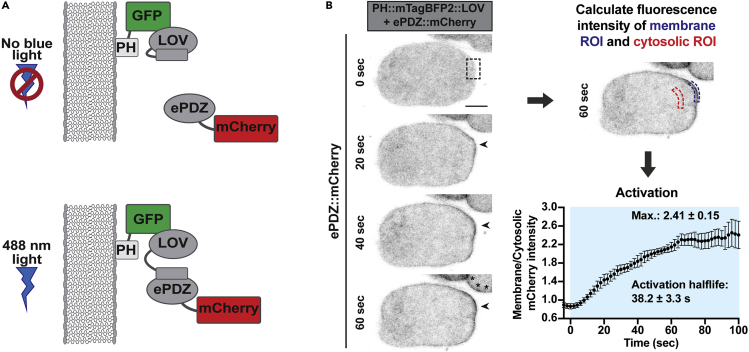
Quantification of light-induced relocalization (A) Scheme for the induced recruitment of ePDZ::mCherry to membrane-bound PH::eGFP::LOV. (B) Time-lapse images of a *C. elegans* zygote with membrane-bound PH::eGFP::LOV (not shown) and cytosolic ePDZ::mCherry, with local LOVpep activation (box). Arrow points to membrane recruitment of ePDZ::mCherry, asterisks indicate LOVpep activation outside the ROI in a neighboring embryo, likely due to light scattering. Calculating the ratio of fluorescence intensity in the activated membrane ROI and a cytosolic ROI of equal (or comparable) size allows to determine the maximum intensity ratio and activation half-life. LOVpep was activated using a 488 nm laser line with laser intensity at 0.01% and pixel dwell time of 8 ms, started at 0 s and was applied in between each time point (2 s interval). Anterior side of the zygote is to the left. Scale bar, 10 μm. Data are mean ± SEM (n = 4).
    Methods video S1. Representative time-lapse video of an embryo expressing membrane-bound PH::GFP::LOV (not shown) and cytosolic ePDZ::mCherry (white), related to Step-by-step method details point 4 The embryo is going through cytokinesis and is exposed to alternating dark and global activating (+488 nm light) conditions. “+488 nm” indicates that LOVpep was activated using a 488 nm laser line, with laser intensity at 0.001% (0.01 μW) and pixel dwell time of 8 ms, applied in between each time point (5 s interval). Frame rate video 15 fps, anterior to the left.
    Methods video S2. Representative time-lapse video of two zygotes expressing EGFR-TM::mTagBFP2::LOV (not shown) and ePDZ::mCherry (white), related to Step-by-step method details point 4 (A) a zygote without and (B) a zygote with continuous anterior local LOV activation (box), as shown in Figure 6. Both zygotes are staged at the end of meiosis II, which can be seen by the local deformation at the anterior side (anaphase of meiosis II, and formation of the polar body) and subsequent membrane ruffling. LOVpep was continuously activated using a 488 nm laser line, with laser intensity at 0.01% (0.1 μW) and pixel dwell time of 8 ms, applied in between each time point (2 s interval). Frame rate video 20 fps, anterior to the left.
5. Assess for unintended light-induced heterodimerization during sample preparation (Figure 4).
  a. Prepare the sample for imaging, while preventing exposure to environmental blue light as described above.
  b. Locate the sample of interest using transmitted light that has been filtered for blue light and acquire a snapshot of the ePDZ fusion protein.
  c. Excite the entire field of view with 488 nm laser light (similar to the acquisition of an eGFP image) to induce global light-induced heterodimerization and *immediately* acquire a second snapshot of the ePDZ fusion protein.
  d. To obtain a dark-state, non-activated condition, leave the sample on the microscope for approximately 5 min, shielding it from all environmental light. Afterwards, acquire a third snapshot of the mobile ePDZ fusion protein.
  e. Compare the snapshots to determine if unintended light-induced heterodimerization occurs during sample preparation. If needed, quantify the signal intensities of the ePDZ fusion protein outside and inside the area where the LOVpep fusion protein is localized (also see next point for how to quantify) (Troubleshooting 4).

**Figure 4:**
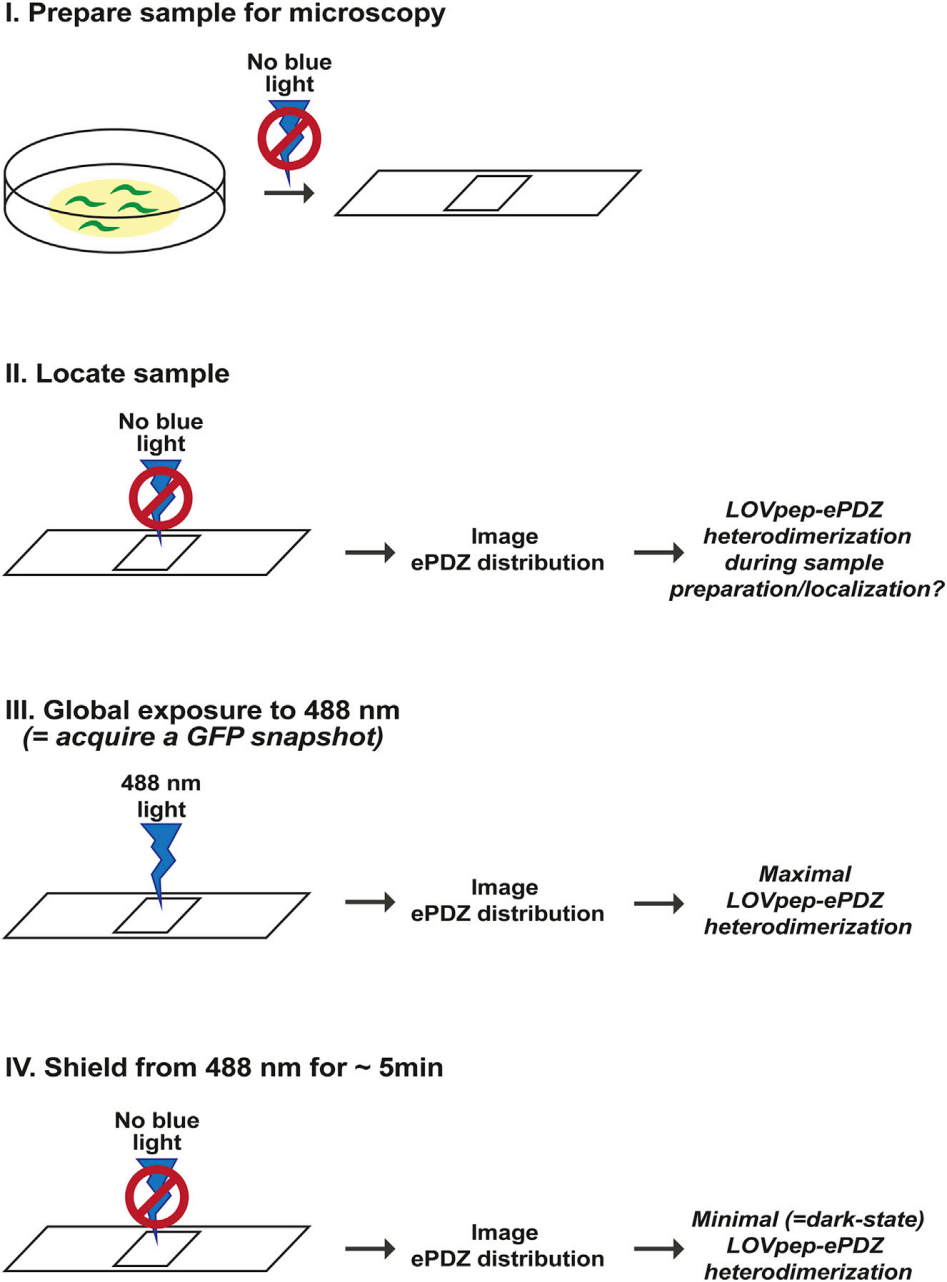
Schematics of the steps to assess for unintended light-induced heterodimerization during sample preparation

***Note:*** Global light-induced heterodimerization refers to applying 488 nm light to the entire cell/ field of view to activate all LOVpep within the sample, whereas local light-induced heterodimerization refers to applying 488 nm light to a defined region of interest (ROI) within the cell to activate LOVpep with subcellular resolution.

**Figure 5:**
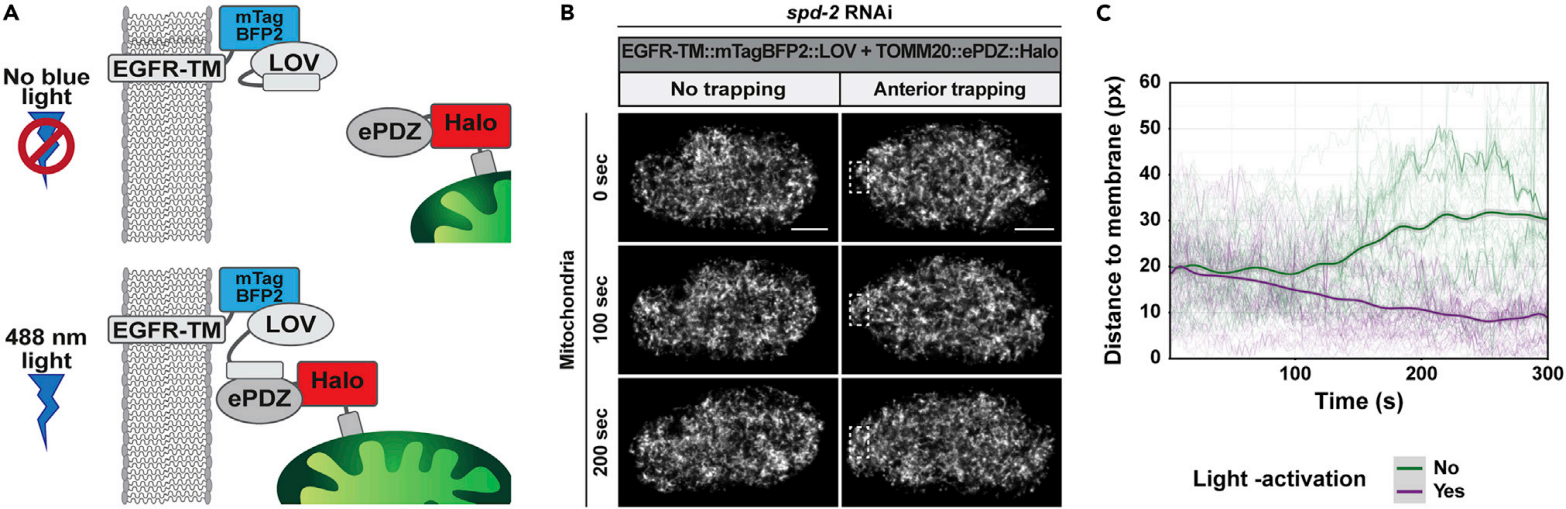
Determining the effect of LOVpep activation on organelle distribution (A) Scheme for the induced recruitment of mitochondria with TOMM20::HALO::ePDZ to membrane EGFR-TM::mTagBFP2::LOV. (B) Representative time-lapse images of *C. elegans* zygotes with membrane EGFR-TM::mTagBFP2::LOV (not shown) and mitochondria with TOMM20::HALO::ePDZ without or with (box) local LOVpep activation. (C) Particle tracking analysis in C. elegans zygotes without or with LOV activation, with tracking performed in the same area as where activating light was used (zygotes with activation) or in a comparable region (zygotes without activation). Solid line is the mean of all tracked particles (n = 4 zygotes each). LOVpep was activated using a 488 nm laser line, with laser intensity at 0.01% and pixel dwell time of 8 ms, started at 0 s and was applied in between each time point (2 s interval). Zygotes were imaged following completion of meiosis II and treated with *spd-2* RNAi to prevent mitochondrial streaming toward the future posterior side. Anterior side of the zygote is to the left. Scale bar, 10 μm. Figure reprinted with permission from De Henau et al. (2020).
  h. Also here, take along a negative control that only expresses the ePDZ fusion construct. No relocation should be observable using the settings used in (g).
    **CRITICAL:** When designing and interpreting experiments keep in mind that also in the dark-state ePDZ will bind to LOVpep, although with an approximately 6-fold lower affinity compared to the photo-activated state (Hallett et al., 2016).
    ***Note:*** Fusing LOVpep to different protein domains might influence activation efficiency or prevent ePDZ binding altogether (Hallett et al., 2016). For example, we needed approximately 10 times more energy to activate LOVpep anchored to the membrane using a transmembrane region (Figure 6), compared to when anchoring it using a pleckstrin homology domain (Figure 3B) (De Henau et al., 2020).
    **Figure 6:**
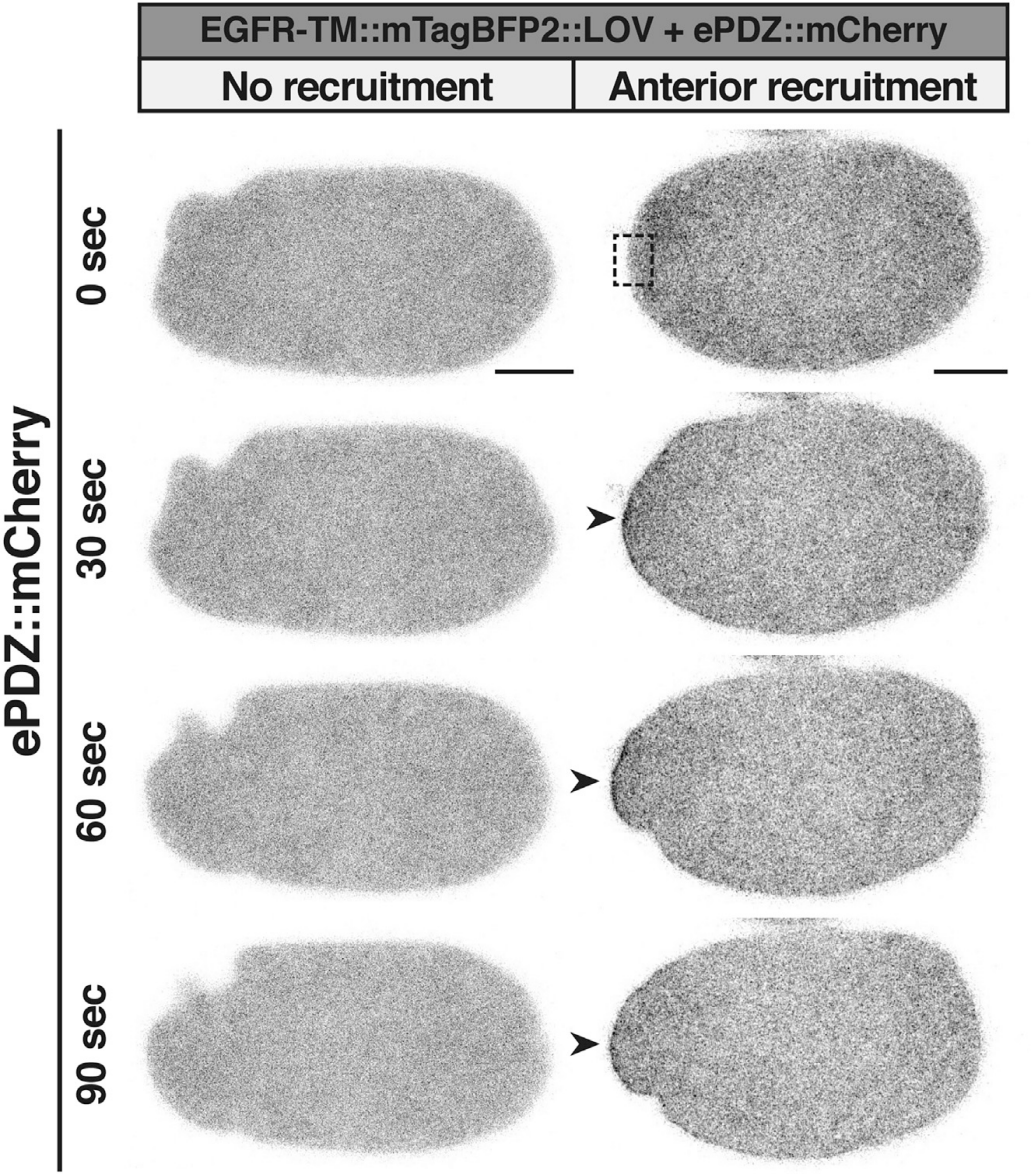
Representative time-lapse images of *C. elegans* zygotes with membrane EGFR-TM::mTagBFP2::LOV (not shown) and cytosolic ePDZ::mCherry without or with LOVpep activation The zygote not exposed to blue light (left column) does not display membrane recruitment of ePDZ::mCherry. The zygote with local LOV activation (right column, box) shows successful local membrane recruitment of ePDZ::mCherry (arrow). LOVpep was activated using a 488 nm laser line, with laser intensity at 0.01% and pixel dwell time of 8 ms, started at 0 s and was applied in between each time point (2 s interval). Anterior side of the zygote is to the left. Scale bar, 10 μm. Figure reprinted with permission from De Henau et al. (2020).

6. Determine 488 nm laser settings for light-induced heterodimerization
  **CRITICAL:** Since LOVpep activation requires very low light exposure, the minimal amount of laser power required for LOVpep activation should be used during experiments. This is especially important for local photoactivation experiments, since excessive laser power combined with light scattering can cause significant activation outside the region of interest. In our hands, an excitation power of 2 iterations of 0.1–0.01 μW 488 nm laser light with a pixel dwell time of 8 μs every 2–5 s (i.e., in between every image acquisition) is sufficient to recruit ePDZ-mCherry to membrane-bound PH-LOV. This amount of excitation power is much lower than what is generally needed to excite and observe eGFP using confocal microscopy. The amount of power needed to maintain activation might increase upon longer time intervals (Hallett et al., 2016).
  a. Starting with a dark-state, non-activated sample, set up a time-lapse experiment for *local* light-induced heterodimerization: excite a ROI with the 488 nm laser in between every acquisition of snapshots of the mobile ePDZ fusion protein. Because the reversion half-life of LOVpep-ePDZ is relatively fast (approximately 50 s) (Fielmich et al., 2018; Hallett et al., 2016), try to keep the time interval in between every 488 nm excitation as short as possible.
  b. Repeat this using different intensities, pixel dwell time, iterations, and time intervals for the 488 nm laser. Since high-intensity blue light may be cytotoxic and causes off-target activation, aim for lower power settings and shorter pixel dwell time and rather increase the number of iterations to achieve light activation. Try to find the lowest/shortest settings for these parameters for which you can still see ePDZ relocation and which are compatible with your experiments.
  c. Take along a negative control that only expresses the ePDZ fusion construct. No relocation or phenotype (e.g., light-induced toxicity) should be observable using the settings from (b).
    ***Optional:*** After acquisition, process the time-lapse images to reduce background noise and segment activated organelle/structure if needed for signal quantification.
  d. Calculate the ratio of fluorescence intensity inside the activated ROI to the intensity in a ROI of similar size outside the area of activation. This analysis produces a maximum intensity ratio as well as the activation half-life (Hallett et al., 2016) (Figure 3B).
  e. With the results from the previous step, determine the minimal amount of power needed to achieve light-induced activation.
    ***Optional:*** Determine how much light-induced heterodimerization occurs surrounding/outside the area of activation and how this would affect your experiment. For example, light-induced activation outside the area of activation can be observed in a neighboring embryo in Figure 3B (bottom image, asterisks), despite using very low levels of 488 nm laser light to activate a defined small area (Troubleshooting 5).
    **CRITICAL:** Because of variation in laser power over time, steps (a)–(g) should be repeated periodically using a reference strain to calibrate the amount of laser power required for local activation of LOVpep prior to experiments.
  f. Using the minimal amount of power for light-induced activation, repeat this procedure with the LOVpep and ePDZ fusion constructs you will use in your experiments.
  g. Determine if continuous, transient, or pulsed light-induced activation is needed for the desired effect on organelle translocation. For example, we used continuous light activation to trap mitochondria at the cell membrane (De Henau et al., 2020) (Figure 5), while Nijenhuis et al. used pulsed activation to explore how organelles recover from light-induced mislocalization (Nijenhuis et al., 2020).
    ***Note:*** The sensitivity and activation and reversion kinetics of LOVpep-ePDZ dimerization following light illumination are important parameters to consider. They determine how often the proteins need to be exposed to blue light in order to induce, maintain, and turn off dimerization to get the desired experimental conditions. Light-induced dimerization of LOVpep-ePDZ occurs with a half-life of approximately 40 s and will revert to its original state in the dark with a reversion half-life of approximately 50 s (Fielmich et al., 2018; Hallett et al., 2016) (Figure 3B). This is relatively fast and in practice means that photoactivation is carried out in between every image acquisition to maintain dimerization.

7. Perform light-induced heterodimerization experiments
  a. Using the settings for 488 nm light-induced activation determined in step 6, set up your imaging parameters.
  b. Locate the sample of interest using transmitted light that has been filtered for blue light.
  c. As noted above, global light-induced heterodimerization can be achieved similar to acquiring GFP images, while local light-induced heterodimerization is achieved by exposing only the region of interest (ROI) with a 488 nm laser.
  d. Afterwards, determine if the light-induced heterodimerization of LOVpep and ePDZ has the desired effect on organelle relocation. For example, to determine if local activation of membrane LOVpep effectively trapped mitochondria labeled with ePDZ in the activated area, we used particle tracking analysis of the mitochondrial signal (Figure 5C) (De Henau et al., 2020). Alternatively, organelle redistribution dynamics can be quantified by comparing changes in total intensity in the activated and non-activated area (Nijenhuis et al., 2020) (Troubleshooting 6).

***Note:*** Trapping of an organelle, for example trapping of mitochondria near the cell membrane (Figure 5) (De Henau et al., 2020), will most likely result in only moderate organelle enrichment at the site of interest. Trapping also requires the organelle of interest to occasionally be present at the site of interest in order for LOV and ePDZ dimerization to be able to occur upon light activation. We therefore prefer to refer to this method as local trapping of an organelle rather than relocation.

## Expected outcomes

Using a strain in which heterodimerization is straightforward to score, such as cytosolic ePDZ and membrane anchored LOVpep, there will be no to minimal dimerization observable in dark state (Figure 6). In such a strain, global light activation will cause a clear and fast dimerization of ePDZ and LOVpep (Methods video S1). Local light activation will cause clear ePDZ-LOVpep dimerization in the activated region, with an expected 2- to 6-fold increase in relative fluorescence intensity of ePDZ in the subcellular location of LOVpep, with lower levels of dimerization observable in the surrounding region (Methods video S2, Figures 3 and 6). Exposure to laser light of 561 nm or above will not cause dimerization, nor exposure to transmitted light passed through an orange optical filter. LOVpep and ePDZ fusion constructs that are expressed individually will not change subcellular distribution upon light activation.

**Figure 7:**
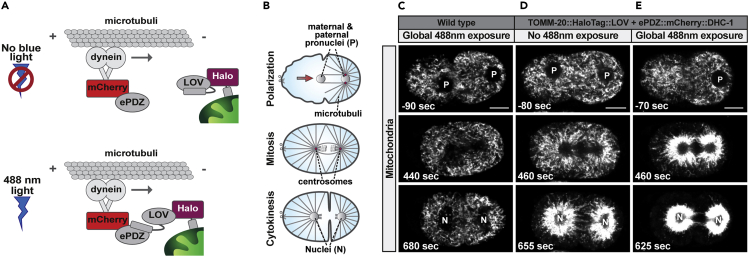
Relocalization of mitochondria fused to LOVpep, with dynein heavy chain fused to ePDZ (A) Scheme for the induced transport of mitochondria with TOMM20::HALO::LOVpep via dynein heavy chain fused to ePDZ and mCherry. (B) Graphical representation of the developmental stages imaged in (C)–(E). Note the development of microtubules during mitosis and cytokinesis. Upon heterodimerization of LOVpep-ePDZ, dynein is expected to transport mitochondria along these microtubules in retrograde direction, toward the centrosomes and (pro)nuclei. (C–E) (C) Mitochondrial dynamics in WT embryos that do not express LOVpep and ePDZ, and (D–E) in embryos that express dynein heavy chain fused to ePDZ (epdz::mcherry::dhc-1) and mitochondria labeled with LOVpep. Embryos in (C) and (E) were exposed to 488 nm laser light, with laser intensity at 0.001% and pixel dwell time of 8 ms, applied in between each time point (5 s interval). The embryo in (D) was imaged under the same conditions but with the 488nm laser shut off and shielded from all environmental blue light. Note that mitochondria relocate toward the center of the cell (= toward the (pro)nuclei), in a moderate manner in (D) and an even more pronounced manner in (E). Anterior side of the embryos is to the left, t(0) = contact between maternal and paternal pronuclei. Mitochondria were visualized using Mitotracker Deep Red FM (Thermo Fisher Scientific).

Using LOVpep-ePDZ for organelle relocation will cause moderate to strong redistribution of the tagged organelle, depending on the cellular machinery that is used (and available) for relocation (Figure 7, compare panel C and E, Methods videos S3, S4, and S5).

Methods video S3. Representative time-lapse video showing mitochondrial dynamics in a wild-type embryo (i.e., not expressing ePDZ or LOVpep), corresponding to Figure 7C, and related to Expected outcomes Mitochondria were visualized using Mitotracker Deep Red FM (Thermo Fisher Scientific). The embryo was exposed to 0.001% 488 nm laser light (0.01 μW) and pixel dwell time of 8 ms, applied in between each time point (5 s interval). Frame rate video 30 fps, anterior to the left, t(0) = contact between maternal and paternal pronuclei.

Methods video S4. Representative time-lapse video showing mitochondrial dynamics in an embryo expressing dynein heavy chain fused to ePDZ (epdz::mcherry::dhc-1) and mitochondria labeled with LOVpep, corresponding to Figure 7D, and related to Expected outcomes Mitochondria were visualized using Mitotracker Deep Red FM (Thermo Fisher Scientific). The embryo was shielded from all environmental blue light during imaging. Frame rate video 30 fps, anterior to the left, t(0) = contact between maternal and paternal pronuclei.

Methods video S5. Representative time-lapse video showing mitochondrial dynamics in an embryo expressing dynein heavy chain fused to ePDZ (epdz::mcherry::dhc-1) and mitochondria labeled with LOVpep, corresponding to Figure 7E, and related to Expected outcomes Mitochondria were visualized using Mitotracker Deep Red FM (Thermo Fisher Scientific). The embryo was exposed to 0.001% 488 nm laser light (0.01 μW) and pixel dwell time of 8 ms, applied in between each time point (5 s interval). Frame rate video 30 fps, anterior to the left, t(0) = contact between maternal and paternal pronuclei.

## Limitations

Blue-light-induced LOVpep-ePDZ dimerization is a powerful technique that allows to address the importance of protein and organelle positioning. This technique is applicable to many organisms, and the single wavelength of light necessary to manipulate their dimerization makes for a simple experimental setup. However, newly designed constructs for photo-inducible heterodimerization do not always result in the desired organelle manipulation and/or can show unwanted dark-state binding, and it is likely that alternative organelle adaptors, proteins used for relocalization, LOV-ePDZ variants with altered affinity (Strickland et al., 2012) or even other light-inducible heterodimerization systems need to be tested. In addition, LOVpep does not tolerate C-terminal fusions, posing a problem to directly label a number of organelle adaptors(van Bergeijk et al., 2015). It is therefore recommended to combine the technique of light-inducible heterodimerization with an efficient and fast cloning approach to be able to rapidly test alternative fusion constructs.

Given the high sensitivity of the LOVpep-ePDZ system, preventing unwanted activation by environmental light or light scattering during culturing and during imaging can be difficult (see for example Figure 3B, asterisks). Activation is also caused by imaging fluorescent proteins that have spectral overlap with the LOVpep domain, such as mTagBFP2 and Venus. Combining the LOVpep-ePDZ system with multiple fluorescent proteins can therefore become challenging and requires careful controls for aberrant activation. In addition, also in the dark-state ePDZ will bind to LOVpep, and while this is with an approximately 6-fold lower affinity compared to the photo-activated state (Hallett et al., 2016), this binding might be sufficient to cause unwanted organelle translocation and severely affect experimental analysis. Dark-state activation is for example observed in Figure 7, in which a combination of mitochondrial targeted LOVpep combined with ePDZ fused to dynein heavy chain DHC-1 causes considerable mitochondrial translocation toward the (pro)nuclei even in dark-state conditions, likely due to constitutive recruitment of ePDZ to LOVpep (compare Figures 7C–7E) (Troubleshooting 7).

LOVpep-ePDZ dimerization following light illumination has a relatively high activation and reversion half-life. While these properties allow to control organelle position with high spatial and temporal resolution, they also impose that the system needs to be continuously illuminated with blue light if stable activation is desired. This could be technically challenging to combine with fast live imaging and might potentially induce phototoxicity in long-term imaging sessions.

Finally, as discussed earlier, several light-inducible protein dimerization techniques have been developed, each with their own sensitivity and dynamic range. The choice of system to use will ultimately depend on the requirements for speed of activation, reversibility, and depth of tissue to be accessed. It is therefore recommended to understand the advantages of each system before deciding which one to use (Hallett et al., 2016; Nijenhuis et al., 2020; van Bergeijk et al., 2015).

## Troubleshooting

### Problem 1

No colonies after transformation of the SapTrap assembly

### Potential solution

Make sure all the SapI cleavage sites are compatible. Make sure to resuspend SapI before pipetting, SapI is prone to precipitation. Make sure to use ATP and not dATP.

### Problem 2

Many colonies with no insert after transformation of the SapTrap assembly

### Potential solution

Make sure the last 37°C cutting step is at least 1 h. In addition, place the reaction tube immediately on ice after the SapTrap assembly protocol is finished. Leaving it at room temperature (18°C–25°C) allows re-ligation of the cut empty pXF87 backbone.

### Problem 3

No expression of a transgene construct designed for germline expression

### Potential solution

Verify that the construct has no internal stop codons or design flaws, for example by expressing it in non-germline tissues. If the construct is properly expressed in non-germline tissues, it likely suffers from germline silencing when targeted to the germline. To circumvent silencing, optimize the sequence for germline expression (Fielmich et al., 2018), remove homology to piRNAs (Batista et al., 2008) and/or introduce PATC introns (Frokjaer-Jensen et al., 2016).

### Problem 4

Significant unintended light-induced heterodimerization during sample preparation

### Potential solution

Make sure to work in a closed room so all environmental light that might reach the sample can be controlled. Ideally, perform sample preparation and imaging in the same room to avoid light exposure in between these two steps.

### Problem 5

Significant light-induced heterodimerization outside the activated ROI

### Potential solution

Lower the exposure to the activating 488 nm laser to reduce light scattering. In addition, consider if the mobility of the tags used to anchor LOVpep might explain diffusion of activated heterodimers outside the ROI and potentially replace these with tags with lower mobility.

### Problem 6

No light-induced heterodimerization or organelle translocation

### Potential solution

Analyze if LOVpep and ePDZ in the fusion constructs are still functional, by combining them with characterized and suitable complementary constructs. For example, to know if an organelle targeted LOVPEP fusion construct is functional, combine it with a cytoplasmic ePDZ and determine if ePDZ relocates to the organelle after light activation.

If both constructs are functional but no organelle translocation is observed, make sure that the constructs effectively overlap in subcellular location and are able to interact. When motor proteins are used, make sure that they are active at the time of light activation and that the cytoskeletal network that is needed for translocation is available.

### Problem 7

Heterodimerization or organelle translocation is observed in dark-state conditions.

### Potential solution

Firstly, make sure that the dark-state condition lacks all forms of blue light, by using filters that block blue light in the dissection scope and microscope, illuminating the working room with red light instead of white light, turning off blue light emission from computer monitors, etc.

Secondly, the binding affinity of LOVpep and ePDZ in dark-state can be sufficient to cause constitutive and unwanted organelle translocation even in the absence of all forms of blue light. As mentioned above, we observed a clear example of dark-state mitochondrial translocation when using a combination of mitochondrial ePDZ and LOVpep fused to dynein heavy chain DHC-1 (Figure 7D).

Reducing dark-state activation might be achieved by lowering expression levels of both constructs or of the LOVpep containing construct in case overexpression constructs are used (Nijenhuis et al., 2020). Fusing alternative motor proteins to LOVpep could also help to reduce dark-state activation. A noteworthy example here is the development of a photosensitive kinesin that is activated upon blue light. This adds a second layer of light-sensitive control and effectively reduced dark-state activation(Nijenhuis et al., 2020).

Finally, mutants of LOVpep or ePDZ with lower dark-state binding affinity (Strickland et al., 2012), or other light-inducible heterodimerization systems with lower dark-state binding affinity, such as the milli variant of the iLID-SspB system(Zimmerman et al., 2016), could help reduce dark-state organelle translocation.

## Resource availability

### Lead contact

Further information and requests for resources and reagents should be directed to and will be fulfilled by the Lead Contact, Sasha De Henau (sasha.dehenau@gmail.com).

### Materials availability

This study did not generate any unique materials or reagents.

### Data and code availability

This study did not generate any unique datasets or code.

## References

[bib1] Adrian M., Nijenhuis W., Hoogstraaten R.I., Willems J., Kapitein L.C. (2017). A phytochrome-derived photoswitch for intracellular transport. ACS Synth. Biol..

[bib2] Batista P.J., Ruby J.G., Claycomb J.M., Chiang R., Fahlgren N., Kasschau K.D., Chaves D.A., Gu W., Vasale J.J., Duan S. (2008). PRG-1 and 21U-RNAs interact to form the piRNA complex required for fertility in *C. elegans*. Mol. Cell.

[bib3] Berkowitz L.A., Knight A.L., Caldwell G.A., Caldwell K.A. (2008). Generation of stable transgenic *C. elegans* using microinjection. J. Vis. Exp..

[bib4] Bugaj L.J., Choksi A.T., Mesuda C.K., Kane R.S., Schaffer D.V. (2013). Optogenetic protein clustering and signaling activation in mammalian cells. Nat. Methods.

[bib5] De Henau S., Pages-Gallego M., Pannekoek W.J., Dansen T.B. (2020). Mitochondria-derived H_2_O_2_ promotes symmetry breaking of the *C. elegans* zygote. Dev. Cell.

[bib6] Engler C., Gruetzner R., Kandzia R., Marillonnet S. (2009). Golden gate shuffling: a one-pot DNA shuffling method based on type IIs restriction enzymes. PLoS One.

[bib7] Fan X., Henau S., Feinstein J., Miller S.I., Han B., Frokjaer-Jensen C., Griffin E.E. (2019). SapTrap assembly of *Caenorhabditis elegans* MosSCI transgene vectors. G3 (Bethesda).

[bib8] Fay D. (2006). Genetic mapping and manipulation: chapter 1--Introduction and basics. WormBook.

[bib9] Fielmich L.E., Schmidt R., Dickinson D.J., Goldstein B., Akhmanova A., van den Heuvel S. (2018). Optogenetic dissection of mitotic spindle positioning in vivo. eLife.

[bib10] Frokjaer-Jensen C., Davis M.W., Ailion M., Jorgensen E.M. (2012). Improved Mos1-mediated transgenesis in *C. elegans*. Nat. Methods.

[bib11] Frokjaer-Jensen C., Davis M.W., Hopkins C.E., Newman B.J., Thummel J.M., Olesen S.P., Grunnet M., Jorgensen E.M. (2008). Single-copy insertion of transgenes in *Caenorhabditis elegans*. Nat. Genet..

[bib12] Frokjaer-Jensen C., Davis M.W., Sarov M., Taylor J., Flibotte S., LaBella M., Pozniakovsky A., Moerman D.G., Jorgensen E.M. (2014). Random and targeted transgene insertion in *Caenorhabditis elegans* using a modified Mos1 transposon. Nat. Methods.

[bib13] Frokjaer-Jensen C., Jain N., Hansen L., Davis M.W., Li Y., Zhao D., Rebora K., Millet J.R.M., Liu X., Kim S.K. (2016). An abundant class of non-coding DNA can prevent stochastic gene silencing in the *C. elegans* germline. Cell.

[bib14] Gibson D.G., Young L., Chuang R.Y., Venter J.C., Hutchison C.A., Smith H.O. (2009). Enzymatic assembly of DNA molecules up to several hundred kilobases. Nat. Methods.

[bib15] Grimm J.B., English B.P., Chen J., Slaughter J.P., Zhang Z., Revyakin A., Patel R., Macklin J.J., Normanno D., Singer R.H. (2015). A general method to improve fluorophores for live-cell and single-molecule microscopy. Nat. Methods.

[bib16] Guntas G., Hallett R.A., Zimmerman S.P., Williams T., Yumerefendi H., Bear J.E., Kuhlman B. (2015). Engineering an improved light-induced dimer (iLID) for controlling the localization and activity of signaling proteins. Proc. Natl. Acad. Sci. U S A.

[bib17] Hallett R.A., Zimmerman S.P., Yumerefendi H., Bear J.E., Kuhlman B. (2016). Correlating in vitro and in vivo activities of light-inducible dimers: a cellular optogenetics guide. ACS Synth. Biol..

[bib18] Harper S.M., Neil L.C., Gardner K.H. (2003). Structural basis of a phototropin light switch. Science.

[bib19] Kennedy M.J., Hughes R.M., Peteya L.A., Schwartz J.W., Ehlers M.D., Tucker C.L. (2010). Rapid blue-light-mediated induction of protein interactions in living cells. Nat. Methods.

[bib20] Krishnamurthy V.V., Khamo J.S., Mei W., Turgeon A.J., Ashraf H.M., Mondal P., Patel D.B., Risner N., Cho E.E., Yang J. (2016). Reversible optogenetic control of kinase activity during differentiation and embryonic development. Development.

[bib21] Lee S., Park H., Kyung T., Kim N.Y., Kim S., Kim J., Heo W.D. (2014). Reversible protein inactivation by optogenetic trapping in cells. Nat. Methods.

[bib22] Levskaya A., Weiner O.D., Lim W.A., Voigt C.A. (2009). Spatiotemporal control of cell signalling using a light-switchable protein interaction. Nature.

[bib23] Merritt C., Rasoloson D., Ko D., Seydoux G. (2008). 3′ UTRs are the primary regulators of gene expression in the *C. elegans* germline. Curr. Biol..

[bib24] Nance J., Frokjaer-Jensen C. (2019). The *Caenorhabditis elegans* transgenic toolbox. Genetics.

[bib25] Nijenhuis W., van Grinsven M.M.P., Kapitein L.C. (2020). An optimized toolbox for the optogenetic control of intracellular transport. J. Cell Biol..

[bib26] Pathak G.P., Strickland D., Vrana J.D., Tucker C.L. (2014). Benchmarking of optical dimerizer systems. ACS Synth. Biol..

[bib27] Redemann S., Schloissnig S., Ernst S., Pozniakowsky A., Ayloo S., Hyman A.A., Bringmann H. (2011). Codon adaptation-based control of protein expression in *C. elegans*. Nat. Methods.

[bib32] Schneider C.A., Rasband W.S., Eliceiri K.W. (2012). NIH Image to ImageJ: 25 years of image analysis. Nat. Methods.

[bib28] Strickland D., Lin Y., Wagner E., Hope C.M., Zayner J., Antoniou C., Sosnick T.R., Weiss E.L., Glotzer M. (2012). TULIPs: tunable, light-controlled interacting protein tags for cell biology. Nat. Methods.

[bib29] van Bergeijk P., Adrian M., Hoogenraad C.C., Kapitein L.C. (2015). Optogenetic control of organelle transport and positioning. Nature.

[bib30] Walhout A.J., Temple G.F., Brasch M.A., Hartley J.L., Lorson M.A., van den Heuvel S., Vidal M. (2000). GATEWAY recombinational cloning: application to the cloning of large numbers of open reading frames or ORFeomes. Methods Enzymol..

[bib31] Zimmerman S.P., Hallett R.A., Bourke A.M., Bear J.E., Kennedy M.J., Kuhlman B. (2016). Tuning the binding affinities and reversion kinetics of a light inducible dimer allows control of transmembrane protein localization. Biochemistry.

